# Examining Bone, Muscle and Fat in Middle-Aged Long-Term Endurance Runners: A Cross-Sectional Study

**DOI:** 10.3390/jcm9020522

**Published:** 2020-02-14

**Authors:** Ulrike H. Mitchell, Bruce Bailey, Patrick J. Owen

**Affiliations:** 1 Department of Exercise Sciences, Brigham Young University, Provo, UT 84602, USA; bruce_bailey@byu.edu; 2 Spine Research Group, Institute for Physical Activity and Nutrition, School of Exercise and Nutrition Sciences, Deakin University, Geelong 3220, Victoria, Australia; p.owen@deakin.edu.au

**Keywords:** exercise, body composition, adipose tissue, healthy aging, running

## Abstract

Aerobic exercise training has many known cardiovascular benefits that may promote healthy aging. It is not known if long-term aerobic exercise training is also associated with structural benefits (e.g., lower fat mass, higher areal bone mineral density (BMD) and greater muscle mass). We evaluated these parameters in middle-aged long-term endurance runners compared to sex-, age-, height-, and weight-matched non-running controls. Total and regional lean and fat mass and areal BMD were assessed by dual-energy X-ray absorptiometry. Sagittal magnetic resonance images captured the cross-sectional area and thickness of the lumbar multifidus. Runners (*n* = 10; all male) had a mean (standard deviation; SD) age of 49 (4) years, height of 178.9 (4.9) cm, weight of 67.8 (5.8) kg, body mass index (BMI) of 21.4 (1.4) kg/m^2^ and had been running 82.6 (27.9) km/week for 23 (13) years. Controls (*n* = 9) had a mean (SD) age of 51 (5) years, height of 176.0 (5.1) cm, weight of 72.8 (7.1) kg, and BMI of 23.7 (2.1) kg/m^2^. BMI was greater in controls (*p* = 0.010). When compared to controls on average, runners had a 10 percentage-point greater total body lean mass than controls (*p* = 0.001) and 14% greater trunk lean mass (*p* = 0.010), as well as less total body (8.6 kg; *p* < 0.001), arm (58%; *p* = 0.002), leg (52%; *p* < 0.001), trunk (73%; *p* < 0.001), android (91%; *p* < 0.001), and gynoid fat mass (64%; *p* < 0.001). No differences were observed between groups for BMD outcomes or multifidus size. These results underscore the benefits of endurance running to body composition that carry over to middle-age.

## 1. Introduction

As the number of older people in the world rapidly increases, determining lifestyle behaviors that may positively impact health are warranted [1]. Regular aerobic exercise training, such as running, has been touted as having a ‘multi-system anti-aging effect’ [1], capable of improving a range of health-related outcomes associated with chronic diseases [2], such as those underscoring cardiometabolic risk [3]. Potential explanations for these overall benefits of aerobic exercise training may, in part, stem from training-induced increases in lean mass and decreases in fat mass [4,5], as well potential increases in bone mineral density (BMD) [6,7]. However, there is limited evidence to date supporting that these benefits of aerobic exercise training are also seen in middle-aged adults. 

In clinical practice, body composition is commonly measured with dual energy X-ray absorptiometry (DXA) in part due to accessibility, ease of analyses and lower radiation dose when compared to computed tomography (CT) [8]. DXA can measure total body and regional fat mass with high precision (CV of 0.9%–4.4%) [9] and correlation compared to magnetic resonance imaging (MRI; r = 0.990) and CT (r = 0.979) at the same sites [10]. While total body fat percentage is a robust measure of relative adiposity and energy stores [11], patterns of fat distribution (e.g., android and gynoid) appear more important when assessing the health of an individual. For example, excess visceral fat (i.e., that accumulated in the android region) was identified as a risk factor for coronary heart disease and metabolic syndrome [12,13] and android to gynoid fat mass ratio was shown to better predict cardiometabolic (CM) risk when compared to general obesity (e.g., body mass index >30 kg/m^2^) [14]. Given that there is evidence that aerobic exercise training can decrease visceral fat stores in general populations [15], it is of value to determine if similar effects are associated with long-term aerobic exercise training in middle-aged adults. 

Lean mass declines with age, with this trend beginning in the fourth decade and accelerating thereafter [16]. Decreases in muscle strength follow and become disproportionately greater than the loss of muscle mass with increasing age [17]. While the loss of muscle mass and strength can be mitigated with regular resistance exercise training [18], currently 50% of those aged 80 years or greater possess clinically low amounts of lean mass [19]. This highlights that preventative practices may be lacking during middle-age. There is less consensus on the effect of aerobic exercise training on age-related loss of lean mass. Of these limited studies, one reported that lean mass, but not total body weight, declined in 140 older adults aged 65–89 years over a three-year period of moderate-to-high intensity aerobic exercise training [20]. Another study found similar aerobic exercise training was an effective modality for increasing lean mass and improving muscle function [21]. When the goal was to decrease body weight, 12 months of aerobic exercise training resulted in preservation of thigh lean mass [22]. Therefore, long-term aerobic exercise training is likely beneficial for muscle strength [23], yet long distance running may lead to decreases in lean muscle tissue mass [24]. A notable limitation of these studies was that they all examined older adults; thus, whether these associations are similar in middle-aged adults is unknown.

Areal BMD is another DXA outcome and often serves to diagnose osteoporosis. BMD directly correlates with bone mass and low BMD is associated with an increased risk of skeletal fracture [25]. Men reach peak BMD in the third decade of life and experience bone loss thereafter [26]. Bone is a metabolically active organ that responds to mechanical loading with remodeling and osteogenesis [27], a principle that was first articulated in 1892 [28]. However, not every type of mechanical loading is equally beneficial. Brief bouts of multidirectional high impact loading that target bones of interest followed by longer recovery periods are more effective in increasing BMD and in enhancing structural properties than repetitive loading [29]. It has been postulated that bone cells become desensitized and that the osteogenic response to loading becomes saturated after a few loading cycles [30]. This phenomenon is known as the ‘diminishing returns’ principle [6]. This supposition has been shown in prior research. For example, sports associated with high magnitude short duration ground reaction forces, such as gymnastics or volleyball, are associated with greater BMD in female athletes compared to swimmers and non-active controls [31]. Specifically, one longitudinal study [32] showed that eight months of gymnastics training increased BMD compared to baseline, whereas no change in BMD was observed following swimming training or control. In contrast to these sports, the evidence regarding BMD and aerobic exercise training (e.g., running) is less conclusive. Female athletes involved in repetitive aerobic/impact sports, such as distance running and cross-country skiing, have shown no difference in BMD compared to athletes involved in higher impact sports, such as gymnastics, or controls [33,34]. Conversely, the results of a five-year longitudinal study [7] showed that older endurance runners of both sexes undergo less age-related bone loss compared to controls. These mixed findings support further examining the relationship between aerobic exercise training and BMD.

To date, most of the research regarding BMD and exercise training has been conducted using an older female population, which likely reflects the prevalence of osteopenia and osteoporosis. We could only find one study [35] that recruited chronically trained and high-mileage middle-aged distance runners and matched their controls by sex, age and weight. Therefore, this demographic appears to represent an understudied population group with regards to these outcomes. 

The purpose of this study was, therefore, to assess body composition and BMD in middle-aged men who have been involved in long-term high-mileage running compared to sex-, age-, height-, and weight-matched non-running participants.

## 2. Experimental Section

Experimental approach to the problem: this study had a cross-sectional design; data collection occurred from March to September 2018. The project identification code (protocol number) was F17528, the study was approved on 15 February 2018 by the Institutional Review Board for Human Subjects at Brigham Young University.

### 2.1. Subjects 

The participants were informed of the benefits and risks of the investigation prior to signing an institutionally approved informed consent document to participate in the study. Members of the running group had to be men, aged 44–62 years; had to have a history of recreational running for >10 years with an average of >50 km/week and no history of regular resistance training. Members in the comparison group (control) had to have performed less than 150 min/week moderate physical activity, walk less than 15 min to or from their place of work and not have performed regular sport or exercise training in the past 10 years. Participants were excluded if they had a history of spinal surgery, history of traumatic injury to the spine, known scoliosis or kyphosis for which prior medical consultation was sought, and being a current or prior smoker. Participants were also excluded based on contraindications for MRI, such as known claustrophobia, metal objects in the body, pacemaker or implantable cardioverter defibrillator. Participants were recruited by word of mouth. After running group participants were enrolled, we recruited an age-, sex- and height-matched control group. The controls were matched in order of priority to height (within 5 cm), age (within three years), weight (within 3 kg) and BMI (within one point). Each subject had to match in at least two of the categories. 

### 2.2. Procedures

#### 2.2.1. DXA

Total and regional lean and fat mass (kg) and total body percent lean and fat mass (%) were assessed by DXA (Lunar iDXA GE Lunar Corp., Madison, WI, USA) and analyzed using enCORE software version 17 (GE Healthcare, Madison, WI, USA) [36,37,38]. The DXA scanner was calibrated every day before any scans were performed using the GE Lunar calibration phantom. Participants were assigned an individual study identifier code which allowed for blinded assessment of all DXA scans. Patient positioning and manual segmentation using custom regions of interest followed previously established protocols [39]. Manual review and adjustments were made by the DXA technician, as needed. The appendicular regions were defined as the tissue distal to a line bisecting the shoulder joint for the upper appendages and bisecting the hip joint for the lower appendages. Appendicular lean mass (ALM) was calculated as the aggregate of lean mass in both arms (kg) plus both legs (kg). Visceral adipose tissue was estimated using the CoreScan option of the enCORE software (GE Healthcare, Madison, WI, USA) [40,41]. Regional scans were performed for BMD assessments of the lumbar spine (L1–L4) and total hip. 

#### 2.2.2. MRI

To quantify muscle morphology on a 3 T Siemens MAGNETOM Tim Trio (Siemens Healthineers, Erlangen, Germany) a T2-weighted sequence (slice thickness 5 mm, TR 3500 ms; TE 99 ms, FOV 280 × 280 mm, voxel size 1.3 × 1.3 × 4 mm^3^) was used with a four-channel flexible coil under the low back to collect 20 sagittal images encompassing the volume of the multifidus from L1 to L5. Data were exported for offline processing. ImageJ 1.48v (http://rsb.info.nih.gov/ij/) was used to measure the multifidus thickness for all lumbar levels. 

After tracing around the multifidus muscle a custom written ImageJ plugin (“ROI Analyzer”; https://github.com/tjrantal/RoiAnalyzer) was used to measure cross-sectional, anterioposterior, and mediolateral area. 

### 2.3. Statistical Analyses 

All analyses were conducted using Stata statistical software version 15 (StataCorp, College Station, TX, USA). Measures were compared between groups by independent *t*-test. Analyses considered MRI outcomes averaged across all lumbar vertebral levels (L2-S1). An alpha-level of 0.05 was adopted for all statistical tests.

## 3. Results

Ten runners and nine controls were included in all analyses, bar MRI-derived multifidus morphology due to missing data (runners: *n* = 9, controls: *n* = 8). Demographical data are shown in Table 1. On average, runners had been running 82.6 (27.9) km/week for 23 (SD: 13, range: 10–39) years.

### 3.1. Body Composition

Body composition (lean and fat mass) are shown in Table 2. Runners had 4.4 kg greater mean total body lean mass than controls, which equated to 10.0 percentage-points greater mean total body percent lean mass, albeit only total body percent lean mass reached statistically significant. Runners also had 14% greater trunk lean mass. Compared to controls on average, runners had less total body (8.6 kg), arm (58%), leg (52%), trunk (73%), android (91%), and gynoid fat mass (64%). Additionally, the android-to-gynoid ratio was lower in runners. 

### 3.2. Lumbar Multifidus

MRI-derived lumbar multifidus morphology is shown in Table 3. No between-group differences were observed.

### 3.3. Bone Mineral Density

BMD variables are displayed in Table 4. No between-group differences were observed.

## 4. Discussion

In this study we compared the body composition and bone density of two groups of middle-aged age-, height-, and weight-matched men that differentiated by their amount of endurance running. Runners had a greater total body (%) and trunk lean mass, as well as lower total body (kg) and regional fat mass. Neither BMD nor multifidus muscle morphology differed between the two groups.

Although there was no significant difference in body mass between the two groups, total body fat mass was approximately two times greater in the control group compared to the runners, which reflected approximately two-fold greater arm, leg, and trunk fat mass. Healthy body fat percentage for a man between the ages of 40 and 59 years is 11.0%–22.9% [42]. It is of interest that, despite matching for proxy markers of height and weight, the runners’ average was well within this range, but the non-runners fell within the lower boundaries of the overweight category (23.0–28.9 cm/kg^2^).

Regional fat depots differed in size and relative contribution in the two groups. For example, controls stored over 5 kg more fat mass in the trunk area compared to the runners, and the non-runners’ android and gynoid fat masses were on average three times and two times higher, respectively, than that of their runner counterparts. Greater trunk fat accumulations in the non-runners suggest that they may have a higher risk for coronary heart disease and metabolic syndrome compared to the endurance runners [12,13].

The gynoid fat mass was approximately 15% in both groups. The android fat mass represented approximately 6% of the total body fat mass in the runners, compared to about 9% in the non-runners. The android-to-gynoid fat mass ratio was lower in runners compared to control. Higher android/gynoid fat mass ratio is associated with greater cardiovascular disease risk factors [43]; however, despite a between-group difference, both ratios were smaller than 0.9 and are thus considered within healthy range [44]. In our study the android fat mass index of runners was less than half of that in runners. Similarly, gynoid fat mass index in runners was approximately half of that in the control. A recent DXA-based cross-sectional analysis of 1133 men (age range, 20–87 years; mean age, 63 years) reported that when compared to men aged 20–30 years, men aged ≥80 years had a 23% greater gynoid fat mass index and a 82% greater android fat mass index [45]. These results suggest that android fat stores increase with age, which may increase cardiometabolic risk [45]. The results of our study show that, compared to non-runners, long time endurance runners have less fat mass in the trunk area, suggesting that they may have a lower cardiometabolic risk.

The appendicular fat distribution in the upper extremity was similar for both groups with approximately 11% of the total body fat mass. In the lower extremity the runners exhibited on average 2.3 kg less leg fat mass compared to the non-runners, yet the fat mass percentage represented 33% and 30% of the total body fat, respectively. While we do not know if this fat is located subcutaneously or intramuscularly, we believe that the relative higher fat content might be a function of the greater need for energy stores. For example, intramyocellular triacylglycerol, or intramuscular fat, is a readily available substrate source used during longer periods of increased energy expenditure, such as during long distance running [46].

Considering that the average total body mass was similar between groups, total body fat is almost twice as high in the controls compared to the runners, and we were not surprised that runners exhibited over 4 kg (10.0 percentage points) greater lean mass. While appendicular lean mass was not different between groups, trunk lean mass was 3.5 kg greater in the runners. This did not reflect our observations for the lumbar multifidus. Thus, other trunk muscles are likely greater in runners compared to controls. We contend that this may be due to differences in the size of the heart and/or abdominal muscles. A study [47] compared heart size of sprinters, endurance runners and sedentary subjects via chest X-ray. They found that the heart size was larger in endurance runners compared to other groups. Endurance runners generally breathe deeper and faster for a long period of time compared to non-exercising individuals. This increased breathing frequency and depth could act as strengthening stimulus to the respiratory muscles. While we did not measure the size of any respiratory muscles, we speculate that each of the three functional groups of respiratory muscles, the diaphragm, the rib cage muscles (e.g., intercostals, the parasternals, the scalene, and the neck muscles), and the abdominal muscles [48] are greater in runners. This could help explain the great discrepancy in trunk lean mass between endurance runners and non-runners.

BMD was not different between the two groups. While the runners had consistently lower values for the lumbar spine and higher values for the femoral neck, none of the differences were statistically different. These results are partially consistent with the results of MacKelvie et al. [35], who found no difference in lumbar spine BMD in male endurance runners ages 40–55 years compared to matched controls. However, the results of that study show significantly higher BMD in the femoral neck of runners compared to their controls, while ours did not. This may be due to the markedly lower femoral neck BMD measures in both runners and controls in the aforementioned study compared to our own.

Several other studies have reported that endurance runners exhibit lower vertebral [49,50,51,52] and femoral neck bone density [51] compared to non-runners. Comparison between those studies and ours are difficult due to a range of methodological differences. For example, the previous studies all examined younger participants. Moreover, one study did not report the years of running history [50], while the remaining studies used participants who had been training for a shorter time [49,51,52]. One study [52] also used male and female runners, while another [51] used only female runners. In addition, one study [49] used dual photon absorptiometry to obtain BMD, while we used DXA. Although there is a good correlation between dual photon absorptiometry and BMD, the measurements are lower with DXA and are therefore recommended to not be compared directly [53].

The reasons for the discrepancies between our findings and the findings of other studies are not completely clear. One could argue that, since our participants were older, it is possible that the benefits of long-term high-mileage running (i.e., less BMD loss) only manifest themselves after >20 years of running. We do not believe that this time frame is necessary to elicit bone changes, especially since it has been shown that bone in healthy younger athletes responds to appropriate stimuli within six [54] and eight months [32], even if the BMD is high initially. It is more likely that long-term running may attenuate age-related BMD loss, which can only become evident as the runner ages.

Our study was strengthened by the use of DXA and MRI analyses, rather than the use of measures that serve as proxies for body composition only (e.g., BMI). Another strength was the matched-nature of controls. Cross-sectional studies, however, have well known limitations. Despite observations that running was associated with better fat mass distribution, we cannot draw a cause and effect conclusion (e.g., running is the cause for better fat distribution). Similarly, we cannot dismiss the possibility that there is reverse causality (e.g., less android fat mass allows people to continue running, while more forces people to stop). Lastly, while we explained why only men were included in this study, these findings may not apply to women.

## 5. Conclusions

Long-term running was associated with a lower total body fat percentage and less total body and regional fat mass, but not with different BMD or multifidus muscle morphology in middle-aged men, when compared to sex-, age-, height- and weight-matched non-running participants. Since body composition is a known risk factor for many age-related diseases, these results underscore the benefits of endurance running that may carry over to middle-age.

## Figures and Tables

**Table 1:** Descriptive statistics of runners (*n* = 10) and controls (*n* = 9).

Variable	Runners (*n* = 9)	Control (*n* = 8)	*t*	*p*-Value
Age, years	49 (4)	51 (5)	−0.8985	0.382
Height, cm	178.9 (4.9)	176.0 (5.1)	1.2134	0.242
Weight, kg	67.8 (5.8)	72.8 (7.1)	−1.6938	0.109
Body mass index, kg/m^2^	21.4 (1.4)	23.7 (2.1)	−2.9082	**0.010**

Data are mean (standard deviation). Bold font: *p* < 0.05.

**Table 2:** Body composition (lean and fat mass) in runners (*n* = 10) and controls (*n* = 9).

Variable	Runners (*n* = 10)	Control (*n* = 9)	*t*	*p*-Value
Fat mass				
Total body, kg	9.9 (3.7)	18.5 (5.1)	−4.2219	**⁢0.001**
Total body, %	17.0 (7.5)	23.8 (6.5)	−2.0916	0.052
Arm, kg	1.1 (0.4)	2.0 (0.7)	−3.6310	**0.002**
Leg, kg	3.3 (1.3)	5.6 (1.4)	−3.8087	**⁢0.001**
Trunk, kg	4.6 (2.1)	9.9 (3.2)	−4.2933	**⁢0.001**
Android, kg	0.6 (0.4)	1.6 (0.6)	−4.2698	**⁢0.001**
Gynoid, kg	1.5 (0.7)	2.9 (0.7)	−4.2038	**⁢0.001**
Android-to-gynoid, ratio	0.379 (0.083)	0.545 (0.122)	−3.4972	**0.003**
Lean mass				
Total body, kg	56.2 (4.9)	51.8 (4.3)	2.0202	0.059
Total body, %	84.8 (5.2)	74.8 (5.2)	3.9713	**0.001**
Appendicular, kg	25.7 (2.3)	24.7 (2.2)	1.0122	0.326
Arm, kg	6.9 (0.9)	6.7 (0.5)	0.3785	0.710
Leg, kg	18.9 (1.8)	17.9 (1.9)	1.1145	0.281
Trunk, kg	27.3 (2.6)	23.8 (2.2)	2.9690	**0.010**

Data are mean (standard deviation). L: lumbar vertebrae. Bold font: *p* < 0.05.

**Table 3:** Multifidus morphology in runners (*n* = 9) and controls (*n* = 8).

Variable	Runners (*n* = 9)	Control (*n* = 8)	*t*	*p*-Value
Cross-sectional area, mm^2^	4788.2 (473.2)	4992.9 (687.2)	−0.7067	0.491
Mean mediolateral thickness, mm	49.6 (7)	49.4 (6.8)	0.6422	0.531
Mean anteroposterior thickness, mm	133.1 (6.4)	144.2 (18.2)	−0.5933	0.563

Data are mean (standard deviation) and average of all lumbar levels.

**Table 4:** Areal bone mineral density and body composition (lean and fat mass) in runners (*n* = 10) and controls (*n* = 9).

Variable	Runners (*n* = 10)	Control (*n* = 9)	*t*	*p*-Value
Areal bone mineral density, g/cm^2^				
L1	1.014 (0.192)	1.102 (0.126)	−1.1588	0.263
L2	1.120 (0.197)	1.220 (0.149)	−1.2484	0.229
L3	1.143 (0.192)	1.268 (0.180)	−1.4505	0.165
L4	1.112 (0.211)	1.230 (0.195)	−1.2594	0.225
L1-L4	1.099 (0.190)	1.209 (0.161)	−1.3438	0.197
Femoral neck	0.984 (0.117)	0.937 (0.095)	0.9501	0.355

Data are mean (standard deviation). L: lumbar vertebrae.
